# Viral Comorbidities Remodel Host Transcriptome and Redox Signaling in an NADPH Oxidase Isoform-Specific Manner

**DOI:** 10.3390/v18050565

**Published:** 2026-05-16

**Authors:** Rashmi K. Ambasta, Suman R. Das

**Affiliations:** 1 Division of Infectious Disease, Department of Medicine, Vanderbilt University Medical Center (VUMC), Nashville, TN 37067, USA; 2 Department of Otolaryngology, Vanderbilt University Medical Center (VUMC), Nashville, TN 37067, USA

**Keywords:** NADPH oxidase, virus, reactive oxygen species, Nox inhibitors, microRNA, redox signaling pathway, viral comorbidities

## Abstract

Viral comorbidities elicit complex host responses by activating redox-sensitive signaling pathways, prominently those regulated by NADPH oxidase (Nox) enzymes. Nox are critical components of host defense, generating reactive oxygen species (ROS) that modulate key cellular signaling cascades. Under normal physiological conditions, Nox activity is tightly controlled; however, viral infections frequently disrupt this regulation, leading to aberrant upregulation of specific Nox isoforms. Elevated expression of individual Nox enzymes has been observed in infections such as influenza A and hepatitis C virus, while simultaneous activation of multiple Nox isoforms occurs in HIV and SARS-CoV infections. Similar patterns of dual or multi-isoform Nox activation are also reported in complex disease states, including diabetes, thrombosis, and fibrosis. MicroRNAs play a crucial role in this process by selectively regulating Nox isoform expression during viral infection, thereby remodeling the host redox environment. Nox-derived ROS influence multiple downstream signaling pathways, including SMAD, MAPK, CXCR-mediated signaling, and the JNK/ERK axis, promoting inflammation and fibrosis that worsen viral disease outcomes. Additionally, several FDA-approved drugs, investigational agents, and microRNA-based therapeutics show promise in modulating Nox activity. Therefore, this article substantiates how viral infections reprogram host transcriptomic and redox signaling networks, contributing to viral pathogenesis and offering potential therapeutic intervention strategies.

## 1. Introduction

The threat of viral pandemics requires a systematic investigation of the proteomic patterns that host cells activate to fight viral infections. Viral infections cause a surge of new transcriptomic and proteomic patterns within cells and the body. One enzyme increased during viral infections is NADPH oxidase (Nox), which produces reactive oxygen species (ROS) [1,2,3,4,5,6]. These ROS can either moderately or intermittently influence signaling pathways or more severely kill pathogens. Alternatively, ROS may impact both host cells and pathogens.

It has been identified that there are different Nox isoforms and various radicals produced that contribute to the overall pool of ROS [7,8,9,10,11,12,13]. The classical form of the enzyme is NOX2, which is found in neutrophils. This enzyme consists of two membrane-bound subunits, Nox2 and p22phox, along with cytosolic subunits p47phox and p67phox [14,15,16,17,18,19,20]. Together with Rac, these cytosolic subunits assemble at the membrane to form an active enzyme that generates radicals. Nox is a multisubunit enzyme that produces reactive oxygen species (ROS) and kills pathogens, including bacteria and viruses. Several viruses have been reported to increase specific Nox isoforms. Viruses such as HIV, HPV, COVID-19, EBV, and AAV [21,22,23,24,25,26,27,28,29] are known to elevate ROS levels.

These viruses enter the human body as pathogens, which are targeted by Nox-mediated radical production and are subsequently destroyed. If the virus evades Nox-mediated neutrophil attack, it can multiply within the host, leading to infection.

Each Nox isoform produces different types and levels of radicals [30,31,32,33]. Currently known Nox isoforms include Nox1, Nox2, Nox3, Nox4, Nox5, and Duox1/2. The classical form of Nox is Nox2, also called gp91 phox, based on its molecular weight and glycoprotein nature. Nox1 has been identified as the oncogenic form, while Nox4 and Nox5 are major sources of ROS in a failing heart [33,34]. Nox isoforms are present in all cell types but are activated by various signals, such as viral infections.

## 2. Structure and Functions of Nox Isoforms

NADPH oxidase consists of two membrane-bound subunits, Nox2 and p22phox. Nox2, also known as gp91phox, is the classical Nox isoform. The known isoforms of Nox2 include Nox1, Nox4, Nox5, Duox1, and Duox2. The domains of Nox2 and its isoforms primarily consist of transmembrane domains and cytosolic domains, which are conserved across Nox1, Nox4, and Nox5. Nox2 features six transmembrane domains at the N-terminal and a Duox-like domain at the C-terminal, along with binding sites for NADPH and FAD. These domains are conserved across Nox1, Nox2, Nox3, Nox4, and Nox5, while the calcium-binding domain is unique to Nox5, Duox1, and Duox2. The peroxidase domain is present only in Duox1 and Duox2. Structurally, Nox1, Nox2, Nox3, and Nox4 are more similar in their transmembrane and C-terminal domains, whereas Nox5, Duox1, and Duox2 share similarities in their calcium-binding domains at the N-terminal, transmembrane domains in the middle, and Nox/Duox-like domains at the C-terminal. All seven Nox isoforms possess transmembrane domains, NADPH-binding sites, FAD-binding sites, and C-terminal Nox/Duox-like domains. Despite their structural homology, these isoforms differ in calcium-binding capacity, the types of radicals they generate, cytosolic partners, and other characteristics. These unique features enable each isoform to perform different functions under various conditions.

To date, several viral infections have been known to trigger Nox isoforms. Some viruses trigger single Nox isoforms, while others trigger dual Nox isoforms. Nox inhibitors have been shown to suppress symptoms of viral infections [35,36,37,38,39,40,41]. Inhibitors of Nox2 include PR-39 (proline-rich antibacterial peptide), Nox2-dstat, and other Nox-based peptides. Nox1 inhibitors include NoxA1ds. Pep1, Pep3, and melittin are inhibitors of Nox5. Nox4 inhibitors include APX-115, and Nox2 inhibitors include DPI [35] and apocynin. Altogether, there are different inhibitors to inhibit these Nox isoforms and their cytosolic partners.

The Nox1 cytosolic partner is NoxO1 (organizer) and NoxA1 (activator) [42,43,44,45,46,47,48,49,50], while the Nox2 cytosolic partners are p47phox and p67phox. The Nox4 regulatory component known to date is only p22phox, and no cytosolic partners are known. The deletion of NoxO1 limits atherosclerosis and has been shown to be a critical player in Nox1-associated physiological functions. Hence, each Nox isoform has either unique cytosolic partners or shared cytosolic partners that regulate their function.

Nox enzymes are widely found across different kingdoms of life, including fungi, plants, and mammals. Mammals exhibit more complex Nox expression patterns, comprising seven Nox isoforms, namely Nox1-5 and Duox1-2 [51,52,53,54,55,56,57,58,59]. The Nox enzyme features a transmembrane domain at the N-terminal and a C-terminal domain that interacts with p22phox. Most Nox isoforms have six transmembrane domains at both the N- and C-termini, which contain binding sites for FAD and NADPH. These isoforms are conserved homologs, sharing similar domains that help regulate their functions. Nox5 is recognized as a gene associated with blood pressure regulation and is linked to calcium-mediated upregulation. Hyperactivation of Nox5 is also connected to cardiovascular disease, kidney injury, and cancer. The constitutive subunit p22phox associates with cytosolic subunits to activate the enzyme.

Nox2 produces superoxide anion with its partners, such as p22phox, p67phox, p47phox, and Rac, while Nox1 produces superoxide anion with its partners, such as p22phox, NoxO1, and NoxA1. Nox4 produces hydrogen peroxide with its partner p22phox. NOX4 primarily generates hydrogen peroxide, whereas NOX1–3, NOX5, and DUOX1–2 predominantly generate superoxide anions [60,61,62,63]. Therefore, we conclude that each isoform generates distinct types and levels of radicals, with either common or unique partners. The unique radicals produced by each Nox may explain its specific function.

## 3. Differential Expression and Regulation of Specific Nox Isoforms in Different Viral Infections

Viruses have distinct tropisms and cause specific effects on human tissues and the body. For example, SARS-CoV-2 (COVID-19) mainly targets the respiratory system. Hepatitis C virus (HCV) affects the liver, leading to chronic hepatitis and possibly cirrhosis. Human immunodeficiency virus (HIV) damages the immune system, especially by depleting CD4+ T cells. Epstein–Barr virus (EBV) is linked to nasopharyngeal carcinoma and other lymphoproliferative disorders. Human papillomavirus (HPV) infects epithelial tissues, particularly the cervix, and is a major cause of cervical cancer. These viral infections [64,65,66] can differently influence the expression of NADPH oxidase (Nox) isoforms, which are crucial in oxidative stress and cell signaling. The specific Nox isoforms activated depend on the virus and the tissue affected, reflecting unique host-pathogen interactions and disease mechanisms [67,68,69,70]. 

**Figure 1:**
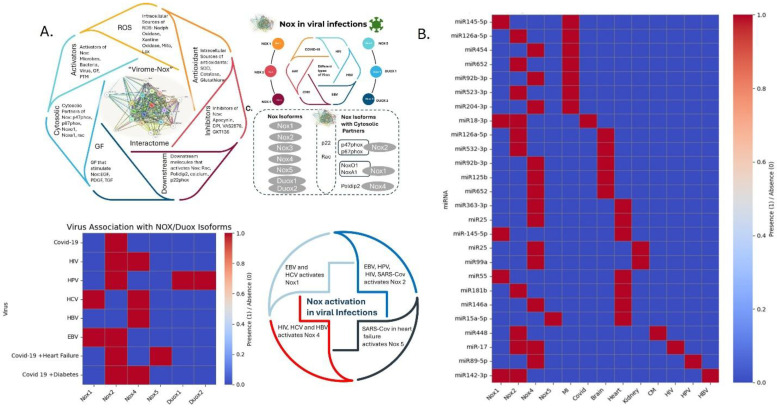
(**A**) **Top:** Schematic illustration showing the role of Nox isoforms in viral infections: ROS-generating enzymes and antioxidants in the cell, along with activators and inhibitors of Nox; growth factors and downstream signaling pathways associated with Nox enzyme; different viruses that elevate Nox isoforms; and the Nox isoforms and their cytosolic partners. (**Down**) Heat map demonstrating the activated Nox isoforms in the presence of different viral infections and types of Nox isoforms in those infections. (**B**) Heat map showing the miR expression associated with different organs and Nox activation. CM: cardiomyopathy; MI: myocardial infarction; HBV: hepatitis B virus.

Heat map analysis (Figure 1) reveals that distinct viral infections induce specific patterns of NADPH oxidase (Nox) isoform expression. The COVID-19 infection upregulates Nox1 and Nox2 [37], while HIV infection increases the expression of Nox1, Nox2, and Nox4 [60]. The HPV infection induces Nox1, Nox2, Duox1, and Duox2 [25]. The other known viral infections, such as HCV, elevate Nox1 and Nox4 [56]. On the other hand, HBV infection triggers Nox1, Nox2, and Nox4 [4]. The EBV infection enhances Nox1 and Nox2 expression [26]. These facts outline the virus-specific regulation of Nox isoforms, suggesting that each pathogen elicits a unique oxidative stress response [70,71,72,73,74,75,76,77,78,79].

In addition, COVID-19 infection in diabetic patients leads to increased expression of Nox2 and Nox4 [73,74], while heart failure conditions in these patients are associated with elevated Nox5 [75,76,77]. The differential expression of Nox isoforms generates distinct reactive oxygen species (ROS), which, in turn, modulate downstream signaling pathways, influence cell fate decisions, and impact disease progression.

Differential viral infections trigger a differential response in host cells, like the COVID-19 virus, which elevates the Nox2 isoform. In patients with COVID-19 who experience heart failure, Nox2, Nox4, and Nox5 are overexpressed. During hyperglycemia in COVID-19 infection, both Nox2 and Nox4 are expressed [78,79]. Furthermore, HIV infection elevates Nox2 and Nox4, while HCV infection increases Nox1 and Nox4. Additionally, HPV infection elevates Nox2, Duox1, and Duox2, while EBV infection elevates Nox1 and Nox2. Therefore, different viral infections elevate distinct Nox isoforms, generating varying levels and types of reactive oxygen species (ROS) and thereby affecting downstream pathways [80,81,82]. HCV infection triggers the expression of both Nox1 and Nox4, while EBV infection induces both Nox1 and Nox2 isoforms. HIV infection elevates Nox2 and Nox4 isoforms, whereas SARS-CoV infection increases only Nox2. Like HIV, HPV infection raises Nox2 and Duox1/2 isoforms. Thus, various Nox isoforms are elevated either in pairs or individually to control viral infections.

## 4. miRNA Regulates the Nox Isoforms Transcriptome in Different Tissues During Comorbid Conditions

MicroRNAs (miRNAs) are crucial regulators of gene expression and influence various Nox isoforms. It has been reported that viruses modulate microRNA to further regulate the expression of Nox isoforms. The important role of miRNA in controlling Nox isoforms has also been observed in HIV and HPV infections. The host cell attempts to combat the virus by triggering different Nox isoforms and increasing radical levels within the cell. Additionally, SARS-CoV infection under hyperglycemic conditions activates both Nox2 and Nox4 isoforms, further complicating signaling pathways in diabetic conditions. In a normal heart, Nox4 expression is regulated by miRNA-363 and miRNA-25 71], while both normal and myocardial infarcted (MI) hearts express Nox1, which is regulated by miRNA-145-5p. In myocardial infarction, Nox2 expression is controlled by miR-126-5p, miR-652, and miRNA-523-3p, whereas Nox4 is regulated by miR-454, miR-92b-3p, and miR-204-3p [72]. Additionally, during cardiomyopathy, miR-448 is expressed [73].

During COVID-19 infection, miR-18-3b regulates Nox2, while miR-17 influences Nox2 and Nox4 expression during HIV infection. Like the heart, the brain expresses different Nox isoforms, including Nox2 and Nox4, which are regulated by miR-126-5p, miR-532-3p, miR-92b-3p, miR-125b, and miR-652. The kidney also expresses Nox4, regulated by miR-25 and miR-99a [77]. Altogether, different tissues express various Nox isoforms, each regulated by specific miRNAs [70,71,72,73,74,75,76,77,78,79]. Similarly, during viral infections, distinct Nox isoforms are expressed and controlled by miRNAs. Since Nox isoforms are crucial for many pathological conditions, inhibiting them might offer a therapeutic option for viral infections.

## 5. Reprogrammed Signaling Pathways During Viral Infections via Nox Isoforms

Hyperglycemia activates Nox2 and Nox4 in patients infected with COVID-19, while only Nox2 is expressed in healthy patients with COVID-19. The virus inhibits the receptors ACE2 and TMPRSS2, which facilitate its entry into the host cell. Consequently, hyperglycemic patients exhibit increased ROS levels, driven by Nox2 and Nox4, leading to abnormalities in downstream signaling pathways. In patients with cardiopathy, Nox4 is considered a new therapeutic target for diabetic vascular complications. Several microRNAs are known to regulate the expression of Nox isoforms in both the presence and absence of the COVID-19 virus.

SARS-CoV infection downregulates ACE2 and upregulates Nox, triggering a cytokine storm in blood vessels and increasing ROS, leading to endothelitis. After endothelitis, thrombosis occurs, leading to a heart attack, and Nox5 has been known to play a critical role in a failing heart. Similarly, HIV-Tat activates two Nox pathways: the Nox4-dependent Ras-ERK pathway and the Nox2-dependent JNK pathway. Each Nox isoform activates distinct signaling pathways and generates different levels of radicals, thereby regulating cell function and disease conditions. The pathways influenced by each Nox isoform differ; therefore, inhibiting these pathways might offer therapeutic potential for various diseases.

**Figure 2:**
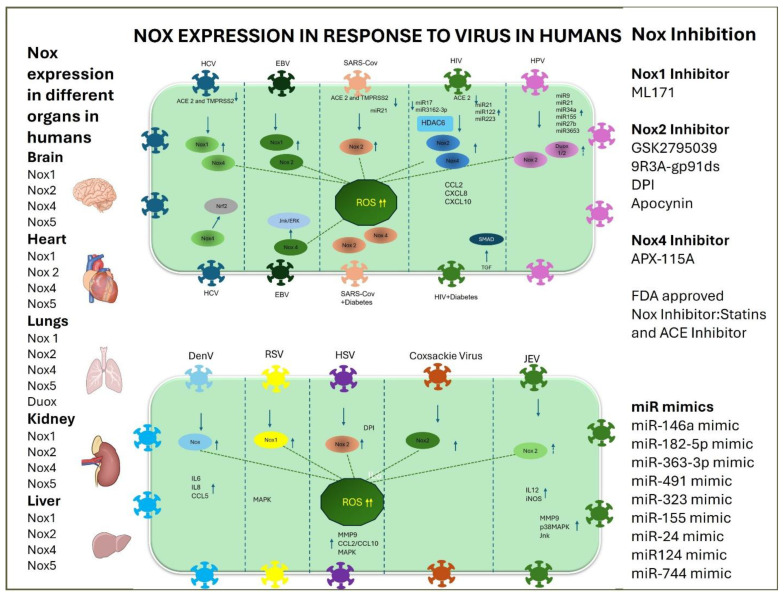
The illustration describes the viral infections and associated complications that trigger different Nox isoforms, along with their signaling pathways. The left-hand list shows the expression of Nox in different organs of the human body, while the list on the right shows the inhibitors and miRNA that inhibit this Nox in an isoform-specific manner. The arrows indicate upstream or downstream regulation.

Viral infections influence tightly regulated signaling pathways in both normal and comorbid conditions through distinct, Nox-specific mechanisms. The HIV virus modulates the p53 and TGF/SMAD pathways in diabetes, while SARS-CoV modulates the NF-kB and TNF/IL pathways, as shown in Figure 2.

The REV affects the TGF and p38MAPK pathways, whereas the JEV alters T cells and increases interleukins. The IAV activates the Nox4-mediated CXCL1/2/10 and CCL3 pathways, while the dengue virus modulates Nox-driven CCL5 and interleukins.

The EBV influences the Nox4-mediated JNK/ERK pathway, while the RSV impacts the Nox and TLR4-mediated p38MAPK pathway, as shown in Table 1.

In summary, different viruses remodel specific cellular signaling pathways that often intersect and influence each other, creating a complex network of dysregulated signals. This intricate interaction ultimately determines key cellular outcomes such as proliferation, apoptosis, and migration. The involvement of multiple NADPH oxidase (Nox) isoforms in these pathways suggests that Nox inhibitors may be potential therapeutic agents. By modulating these signaling cascades, Nox inhibitors could improve patient health, especially in diseased or comorbid conditions.

## 6. Nox-Mediated Viral Disease Outcomes in Different Organs

**Figure 3:**
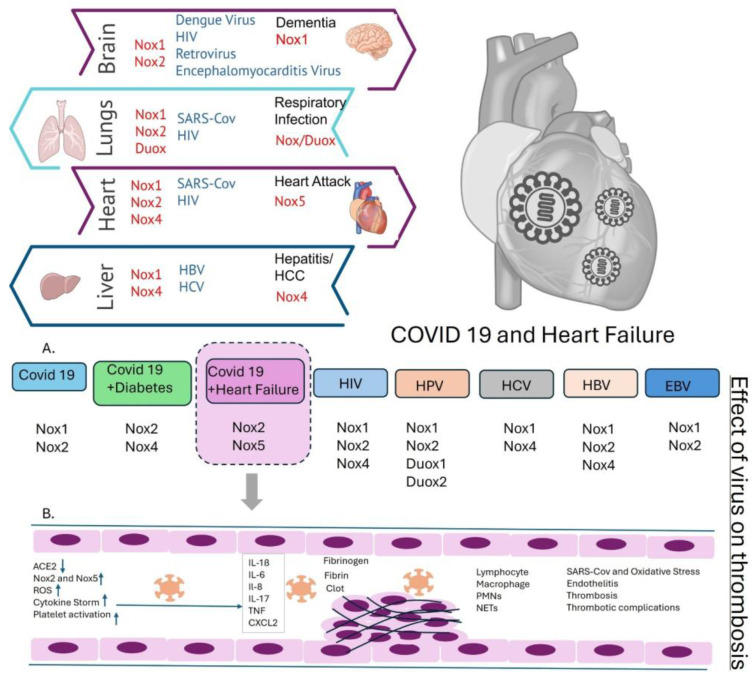
(**Top**) Left: Schematics for the effect of the virus on different organs and thrombotic signaling pathways in the presence of viral infection and Nox activation. Right: heart with virus (**Bottom**) Signaling pathways associated with (**A**) different viral infections and (**B**) Nox isoform activation. The arrow indicates that heat failure with COVID is explained below.

Viral infections can significantly impact multiple organs, including the brain, lungs, and liver. Post-infection complications such as dementia, respiratory infections, and hepatitis have been documented. Notably, different NADPH oxidase (Nox) isoforms are upregulated in these conditions: Nox1 is elevated in dementia, while Nox2 is associated with Alzheimer’s disease. In the lungs, viral infections increase the expression of various Nox isoforms, whereas in the liver, they upregulate Nox1 and Nox4, as shown in Figure 3.

These observations suggest that viral infections trigger distinct patterns of gene regulation and expression across different tissues, influencing the fate of host cells. Therefore, a detailed investigation into these molecular changes could help researchers identify targeted areas for future study and therapeutic development.

## 7. Host Redox Reprogramming by Viral Comorbidities

Viruses reprogram the host cells’ transcriptome in several ways, including NADPH oxidase isoform regulation and redox homeostasis. Redox homeostasis is critical for human health, and a virus may trigger a specific isoform of Nox, thereby disrupting homeostasis and perturbing the normal pathway. This disruption of homeostasis may promote the reprogramming of redox in the host and a differential expression of a specific isoform of Nox, which generates a different type of radicals. These radicals may include superoxide anion, hydrogen peroxide or another type of radical.

This reprogramming is regulated by miRNAs, and different viruses activate different types of miR, which thereby affect a specific Nox isoform; hence, isoform-specific radicals are generated, affecting the redox homeostasis of the cell. Low oxidative stress promotes a healthy cell, while high oxidative stress promotes apoptosis. Intermediate levels of oxidative stress promote signaling pathways in a unique manner. The varying levels of oxidative stress are responsible for the occurrence of viral comorbid conditions.

## 8. Role of Nox Inhibitors in Disease Management of Viral Infections

**Figure 4:**
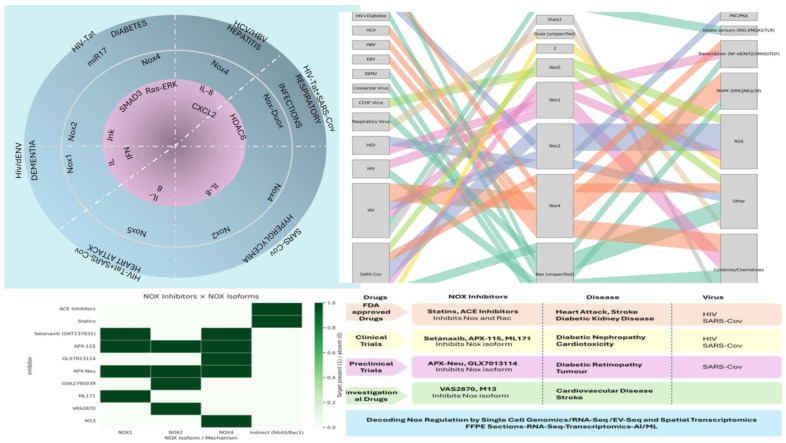
Illustration of Nox signaling and depiction of the list of drugs that inhibit Nox in viral comorbidities, along with the stages of clinical trial phases.

There are various Nox inhibitors that target specific Nox isoforms or broadly inhibit all Nox isoforms. Some well-known Nox inhibitors include DPI (diphenylene iodonium), GKT136901 (Nox1/4 inhibitor), APX-115 (pan-Nox inhibitor), VAS2870 (Nox2/4 inhibitor), and GLX481304 (Nox2/4 inhibitor), as shown in Figure 4. It has been reported that GSK2795029 is used to inhibit Nox2 in H1N1 viral infection, while APX-115A is used to inhibit Nox4 in EBV viral infection (ref). Similarly, Nox1 is inhibited during COVID-19 infection, while DPI is used in HSV infection. Additionally, Apocynin is used to inhibit Nox2. As these inhibitors are specific for either Nox isoforms or broadly inhibit Nox activity, it can be concluded that selective Nox expression is critical for viral infection. Similarly, Nox expression may be modulated by miR mimics.

## 9. miR Mimics Targeting Nox: A Potential Antiviral Therapeutic?

miR mimics can target both Nox and viral infections by modulating Nox gene expression and host immune response. Introduction of synthetic miR mimics that mimic a mature microRNA silences the expression of a particular Nox isoform and thereby alleviates symptoms related to that viral infection. Some of the miR mimics that target Nox include miR652, which silences Nox2 expression in the rat brain and reduces brain damage. miR-146a inhibits Nox4 and protects against myocardial ischemia. Similarly, miR182 suppresses Nox4 and promotes corneal nerve regeneration in diabetic mice; miR-100p targets Nox4 and prevents ischemic brain injury; miR190 attenuates Nox2 and prevents pancreatic cell toxicity in diabetes; and miR-126-5p exerts neuroprotective effects in ischemic stroke by targeting Nox2. There are many miRs that target viral genes like miR24, miR124, miR744, which target MAPK, while miR1388 targets TRAF3. Altogether, miR mimics that target Nox, viruses, or host immune responses, such as cytokines, may offer therapeutic strategies for viral infections. Furthermore, several natural compounds have been identified that can modulate either viral activity or Nox expression, offering additional avenues for treatment. Recently, nanoparticles loaded with siRNA targeting Nox have been used to treat ischemic brain injury.

## 10. Drugs Targeting Nox Undergoing Clinical Trials in Viral Comorbid Conditions

There are different types of drugs that target Nox in viral comorbid conditions. The viral ischemic condition of the heart is known to be treated with FDA-approved drugs like statins and ACE inhibitors. On the other hand, several drugs are undergoing clinical and preclinical trials. The preclinical drugs include GLX7013114, APX-Neu, and GSK2795039, while the clinical drugs include setanaxib, APX-115, and ML171. The potent drugs that target Nox are VAS2870 and M13, as listed in Table 2.

ACE inhibitors and statins inhibit Nox indirectly through the Renin Angiotensin-Aldosterone system (RAAS) and rac respectively, which are known to activate Nox. However, other inhibitors listed in the table above directly inhibit Nox in an isoform-specific manner. Some of them are specific, while others are broad inhibitors of Nox isoforms, also known as pan-Nox or multiple Nox isoform inhibitors. These inhibitors unlock the power of Nox as an antiviral for comorbid conditions.

## 11. Unlocking the Power of the Nox Inhibitors in Viral Comorbidities

Identifying specific Nox isoforms that increase during viral infections is essential for creating targeted protein therapies. Inhibiting these Nox isoforms could reduce symptoms of viral infections and related diseases, such as hyperglycemia and cardiovascular issues. Conditions like diabetes, dementia, hepatitis, respiratory infections, and thrombosis also cause increases in certain Nox isoforms. Therefore, thoroughly identifying each Nox isoform will improve our understanding of the faulty signaling pathways in these illnesses. For example, HIV infection raises Nox1, Nox2, and Nox4 levels, while COVID-19 infection raises Nox1 and Nox2 levels. In diabetic patients with COVID-19, Nox2 and Nox4 are elevated; in heart failure patients with COVID-19, Nox2 and Nox5 are elevated.

Calcium-dependent Nox5 plays a significant role in oxidative stress and MAPK-mediated cardiac hypertrophy. Therefore, inhibiting Nox5 in patients with heart failure may provide relief. Additionally, Nox4 inhibitors have been reported to be beneficial in treating kidney injury, diabetic nephropathy, and cardiovascular diseases. Consequently, future research should focus on identifying and validating specific Nox isoforms as therapeutic targets for viral infections.

## 12. Discussion

Despite several advancements in the field of the mechanisms of viral infections, critical gaps and challenges remain in the identification of hit diagnostic and therapeutic targets. This review is an attempt to identify Nox isoforms that are differentially expressed in various viral infections, and their expression is regulated by specific microRNAs (miRs). The Nox isoform expression is also species-specific, as NOX5 is absent in mice and rats [80]; therefore, findings from preclinical studies using these rodent models may be difficult to reproduce in humans. Consequently, the identification of alternative animal models is essential for preclinical investigations related to NOX5 signaling pathways and the development of NOX5-specific inhibitors. These Nox isoforms are expressed during infection, co-infection and superinfection conditions.

Superinfection can induce virus interaction via different strategies. Both RNA and DNA viruses can regulate superinfection through diverse molecular mechanisms that alter host cell entry pathways and innate immune responses. Viruses such as HIV actively block superinfection using accessory proteins [83] (e.g., Nef and Vpu) that remodel the plasma membrane and downregulate entry receptors like CD4, CCR5, and CXCR4 [81], while heterologous superinfection may still occur depending on receptor usage, target cell type, and immune control. Interferons (IFNs) play a central role in viral interference by suppressing the replication of multiple viruses, including respiratory pathogens such as RSV, influenza virus, rhinovirus, and SARS-CoV-2, although these effects are virus-specific and depend on IFN type, timing, and viral sensitivity; notably, chronic infections like HIV can impair IFN responses, allowing the persistence of multiple viruses. DNA viruses such as HPV also exhibit superinfection exclusion [82], as seen in HPV16-mediated inhibition of HPV18 during viral entry. Together, these examples demonstrate that both homologous and heterologous superinfection exclusion occurs across virus families, shaped by receptor competition, innate immunity, and viral adaptation, with important consequences for pathogenesis and disease severity.

Some DNA viruses, including herpesviruses, can thrive in balanced ROS-rich environments and hence modulate the redox pathway to create a ROS-controlled environment for viral replication. By modulating host redox balance through ROS induction factors possibly encoded within their genome, these viruses reduce oxidative damage to their own genomes and limit ROS-mediated immune activation [84]. Thus, ROS induction in DNA virus infection can be both a host defense and a viral survival mechanism, making it a promising but complex target for antiviral intervention. Consequently, ROS induction represents a complex virus–host interaction and a potential target for antiviral strategies.

Since viral infections activate distinct signaling pathways in a Nox isoform-specific manner, targeted inhibition of these isoforms—either through Nox inhibitors or miR mimics—holds promise as a therapeutic strategy. Future gene expression tools, such as RNA-seq or spatial transcriptomics in viral myocarditis, may offer more therapeutic strategies. This approach could lead to more precise and effective treatments for managing the specific Nox isoforms involved in each viral infection and their systemic complications in the future.

## 13. Conclusions

Evidence indicates that different viruses affect the expression levels of specific isoforms of Nox. This Nox expression is controlled by a reprogrammed transcriptome of miR clusters, which in turn regulates the downstream redox-sensitive signaling pathway. Several Nox inhibitors and miR-based therapies have been reported to provide relief in viral comorbidities. Therefore, we conclude that the virus reprograms the host transcriptome and redox signaling pathway during viral comorbidities.

## 14. Future Directions

Future research in virology is focused on identifying viral mutants and understanding how these variants influence viral comorbidities. The application of artificial intelligence and machine learning for the analysis of viral genomic sequences and their mutations is becoming critical and opens new avenues for precision medicine in virology. In addition, the use of advanced computational approaches in spatial transcriptomics, particularly in FFPE tissues from viral comorbid conditions, provides deeper insights into cell-type-specific responses and multi-omic alterations underlying host–virus pathology. Multiplex cytokine profiling of the serum of affected patients provides insight into the host immune condition and affected cytokines. Identification of uniquely dysregulated genes or cytokines associated with viral comorbidities may ultimately enable more effective and personalized therapeutic strategies for viral infections.

## Figures and Tables

**Table 1:** This table describes the specific Nox isoform upregulated in response to the virus and the signaling pathways.

Virus	Nox	Upstream Signal	Downstream Signal	Reference
HIV + Diabetes	Nox	miR192 and p53	TGF/SMAD3	[23]
HIV	Nox1	CD4+ T cells	IL-1α	[23]
REV	Nox4	TGF	p38MAPK	[28]
JEV	Nox2	Th1 CD4+ and CD8+ T cell	IL12p40 and iNOS	[63]
SARS-CoV	Nox	TLR and miR21; NF-kB	IL-1β/TNFα/IL-8	[59]
SARS-CoV	Nox1 and 2	ACE 2	ROS/TGFß	[22]
SARS-CoV	Nox4	Ang2	Poldip2	[45]
SARS-CoV	Nox2 and Nox5	LV and RV	ROS	[38]
HSV	Nox2	HSK Cornea	ROS	[35]
SARS-CoV	Nox2	Dysregulated miRNA	ROS	[55]
IAV	Nox4		CXCL1/2/10 and CCL3	[5]
DENV	Nox	ROS	IL-6, IL-8, and CCL5	[36]
HIV	Nox	gp120	CXCR5 and CCR3	[24]
IAV	Duox1	ROS	Several cytokines IL	[65]
EBV	Nox4	ROS	Jnk/ERK	[26]
HBV	Nox4	IL8/TNF	CXCL2	[2]
HCV	Nox4	ROS	Nrf2	[76]
HSV	Nox1	PI3K/PKC/ERK1/2/	NF-kB/Nrf2	[39]
Coxsackie Virus	Nox	ROS	Cytokine	[23]
IAV	Nox2	TLR7	PKC	[5]
IAV	Duox2	IFN	Rig1/MDA5	[5]
IAV	Nox1	T Cell	cytokine IL7	[5]
RSV	Nox	TLR4	ERK/p38MAPK	[66]
Respiratory Virus	Nox/Duox	Proinflammatory response		[66]
IAV	Nox4	ROS	MAPK	[5]
CCHF Virus	Nox 5	Protective Nox5 in CCHF patients		[10]

**Table 2:** This table describes the NOX inhibitors as drugs undergoing different stages of clinical trials in viral comorbid conditions.

S No	Nox Inhibitors as Drugs	Stage of Drug Approval	Disease	Virus	Reference
1	ACE Inhibitor	FDA-approved drug	Diabetic Kidney Disease	HIV	[37]
	(Reduces Nox indirectly through RAAS and inhibiting Ang II))				
2	Statins	FDA-approved drug	Heart Attack, Stroke	COVID-19, HIV	[11,33]
	(Inhibits Nox indirectly through rac)				
3	Setanaxib (GKT137831)	Clinical (Phase 1/2)	Diabetic Nephropathy	HIV,	[23]
				COVID-19	
	(Inhibits Nox 1 and Nox 4)		Pulmonary Fibrosis		
			Cholangitis		
			Cardiotoxicity		
			Head and Neck Cancer		
4	APX-115	Clinical (Phase2)	Diabetic Nephropathy	COVID-19	[26]
	(Inhibits Nox1, Nox2, Nox4)		Kidney Injury	EBV	
5	GLX7013114	Preclinical Trial	Diabetic Retinopathy		[12]
	(Nox4-Specific Inhibitor)				
6	GSK2795039	Preclinical	Cardiotoxicity	H1N1	[5]
	(Inhibits Nox 2)				
7	ML171	Clinical	Hyperglycemia/Diabetes	HIV	[23]
	(Inhibits Nox1)		Fibrotic Disease		
8	VAS2870	Potent drug	Cardiovascular Disease	Influenza Virus	[5]
	(Inhibits Nox2, Nox4)		Ischemic Stroke		
9	M13	Potent drug	Ischemia		[68]
	(Inhibits Nox4)		Stroke		

## Data Availability

No new data were created or analyzed in this study. Data sharing is not applicable to this article, as no datasets were generated or analyzed during the current study.

## References

[1] Agarwal S., Sharma H., Chen L., Dhillon N.K. (2020). NADPH oxidase-mediated endothelial injury in HIV- and opioid-induced pulmonary arterial hypertension. Am. J. Physiol. Cell. Mol. Physiol..

[2] Wang H., Wang J., Liu H., Wang X. (2019). [TGF-β1 activates NOX4/ROS pathway to promote the invasion and migration of cervical cancer cells]. Xi Bao Yu Fen Zi Mian Yi Xue Za Zhi.

[3] Smirnova O.A., Ivanova O.N., Bartosch B., Valuev-Elliston V.T., Mukhtarov F., Kochetkov S.N., Ivanov A.V. (2016). Hepatitis C Virus NS5A Protein Triggers Oxidative Stress by Inducing NADPH Oxidases 1 and 4 and Cytochrome P450 2E1. Oxidative Med. Cell. Longev..

[4] Mishra A.K., Hossain M.M., Sata T.N., Pant K., Yadav A.K., Sah A.K., Gupta P., Ismail M., Nayak B., Shalimar Venugopal S.K. (2025). ALR inhibits HBV replication and autophagosome formation by ameliorating HBV-induced ROS production in hepatic cells. Virus Genes.

[5] Hendricks K.S., To E.E., Luong R., Liong F., Erlich J.R., Shah A.M., Liong S., O’Leary J.J., Brooks D.A., Vlahos R. (2022). Endothelial NOX4 Oxidase Negatively Regulates Inflammation and Improves Morbidity During Influenza A Virus Lung Infection in Mice. Front. Cell. Infect. Microbiol..

[6] Du S., Miao J., Zhu Z., Xu E., Shi L., Ai S., Wang F., Kang X., Chen H., Lu X. (2018). NADPH oxidase 4 regulates anoikis resistance of gastric cancer cells through the generation of reactive oxygen species and the induction of EGFR. Cell Death Dis..

[7] El-Wakil D.M., Shaker O.G., Rashwan A.S.S.A., Elesawy Y.F., Samir N. (2024). High-risk human papillomavirus infection and cervical cytopathology: Relationship with cervical nitric oxide levels. Virol. J..

[8] Zhang J., He L., Huang R., Alvarez J.F., Yang D.H., Sun Q., Wang F., Peng Z., Jiang N., Su L. (2023). Synergistic effect of elevated glucose levels with SARS-CoV-2 spike protein induced NOX-dependent ROS production in endothelial cells. Mol. Biol. Rep..

[9] Choi J., Corder N.L., Koduru B., Wang Y. (2014). Oxidative stress and hepatic Nox proteins in chronic hepatitis C and hepatocellular carcinoma. Free Radic. Biol. Med..

[10] Büyüktuna S.A., Doğan H.O., Bakir M., Elaldi N., Gözel M.G., Engin A. (2018). The protective effect and diagnostic performance of NOX-5 in Crimean-Congo haemorrhagic fever patients. J. Med. Microbiol..

[11] DiNicolantonio J.J., McCarty M. (2020). Thrombotic complications of COVID-19 may reflect an upregulation of endothelial tissue factor expression that is contingent on activation of endosomal NADPH oxidase. Open Heart.

[12] de Oliveira A.A., Nunes K.P. (2021). Crosstalk of TLR4, vascular NADPH oxidase, and COVID-19 in diabetes: What are the potential implications?. Vasc. Pharmacol..

[13] Ahn W., Burnett F.N., Pandey A., Ghoshal P., Singla B., Simon A.B., Derella C.C., AAddo S., Harris R.A., Lucas R. (2024). SARS-CoV-2 Spike Protein Stimulates Macropinocytosis in Murine and Human Macrophages via PKC-NADPH Oxidase Signaling. Antioxidants.

[14] Liang S., Liu A., Liu Y., Wang F., Zhou Y., Long Y., Wang T., Liu Z., Ren R., Ye R.D. (2024). Structural basis for EROS binding to human phagocyte NADPH oxidase NOX2. Proc. Natl. Acad. Sci. USA.

[15] Kuihon S.V.N.P., Sevart B.J., Abbey C.A., Bayless K.J., Chen B. (2024). The NADPH oxidase 2 subunit p47^phox^ binds to the WAVE regulatory complex and p22^phox^ in a mutually exclusive manner. J. Biol. Chem..

[16] Treuer A.V., Faúndez M., Ebensperger R., Hovelmeyer E., Vergara-Jaque A., Perera-Sardiña Y., Gutierrez M., Fuentealba R., González D.R. (2023). New NADPH Oxidase 2 Inhibitors Display Potent Activity against Oxidative Stress by Targeting p22^phox^-p47^phox^ Interactions. Antioxidants.

[17] Paclet M.H., Laurans S., Dupré-Crochet S. (2022). Regulation of Neutrophil NADPH Oxidase, NOX2: A Crucial Effector in Neutrophil Phenotype and Function. Front. Cell Dev. Biol..

[18] Nauseef W.M. (2019). The phagocyte NOX2 NADPH oxidase in microbial killing and cell signaling. Curr. Opin. Immunol..

[19] Raad H., Derkawi R.A., Tlili A., Belambri S.A., Dang P.M., El-Benna J. (2019). Phosphorylation of gp91^phox^/NOX2 in Human Neutrophils. Methods Mol. Biol..

[20] Nauseef W.M., Clark R.A. (2019). Intersecting Stories of the Phagocyte NADPH Oxidase and Chronic Granulomatous Disease. Methods Mol. Biol..

[21] Fink B., Hunter J.M., Pietrzkowski Z., Fink R., Brunssen C., Morawietz H., Nemzer B. (2025). A Plant-Based Dietary Supplement Exhibits Significant Effects on Markers of Oxidative Stress, Inflammation, and Immune Response in Subjects Recovering from Respiratory Viral Infection: A Randomized, Double-Blind Clinical Study Using Vitamin C as a Positive Control. Int. J. Mol. Sci..

[22] Chowdhury B., Sahoo B.M., Jena A.P., Hiramani K., Behera A., Acharya B. (2024). NOX-2 Inhibitors may be Potential Drug Candidates for the Management of COVID-19 Complications. Curr. Drug Res. Rev..

[23] Kress T.C., Barris C.T., Kovacs L., Khakina B.N., Jordan C.R., Bruder-Nascimento T., Stepp D.W., MacArthur R., Patel V.S., Chen J. (2025). CD4^+^ T Cells Expressing Viral Proteins Induce HIV-Associated Endothelial Dysfunction and Hypertension Through Interleukin 1α-Mediated Increases in Endothelial NADPH Oxidase 1. Circulation.

[24] Smith L.K., Babcock I.W., Minamide L.S., Shaw A.E., Bamburg J.R., Kuhn T.B. (2021). Direct interaction of HIV gp120 with neuronal CXCR4 and CCR5 receptors induces cofilin-actin rod pathology via a cellular prion protein- and NOX-dependent mechanism. PLoS ONE.

[25] Marullo R., Werner E., Zhang H., Chen G.Z., Shin D.M., Doetsch P.W. (2015). HPV16 E6 and E7 proteins induce a chronic oxidative stress response via NOX2 that causes genomic instability and increased susceptibility to DNA damage in head and neck cancer cells. Carcinogenesis.

[26] Hong S.W., Noh M.H., Kim Y.S., Jin D.H., Moon S.H., Yang J.W., Hur D.Y. (2020). APX-115A, a pan-NADPH Oxidase Inhibitor, Induces Caspase-dependent Cell Death by Suppressing NOX4-ROS Signaling in EBV-infected Retinal Epithelial Cells. Curr. Eye Res..

[27] Ding Y., Zhu W., Sun R., Yuan G., Zhang D., Fan Y., Sun J. (2015). Diphenylene iodonium interferes with cell cycle progression and induces apoptosis by modulating NAD(P)H oxidase/ROS/cell cycle regulatory pathways in Burkitt’s lymphoma cells. Oncol. Rep..

[28] Agawa H., Ikuta K., Minamiyama Y., Inoue M., Sairenji T. (2002). Down-regulation of spontaneous Epstein-Barr virus reactivation in the P3HR-1 cell line by L-arginine. Virology.

[29] Choi J.Y., Byeon H.W., Park S.O., Uyangaa E., Kim K., Eo S.K. (2024). Inhibition of NADPH oxidase 2 enhances resistance to viral neuroinflammation by facilitating M1-polarization of macrophages at the extraneural tissues. J. Neuroinflam..

[30] Pecchillo Cimmino T., Ammendola R., Cattaneo F., Esposito G. (2023). NOX Dependent ROS Generation and Cell Metabolism. Int. J. Mol. Sci..

[31] Zhao G.J., Zhao C.L., Ouyang S., Deng K.Q., Zhu L., Montezano A.C., Zhang C., Hu F., Zhu X.Y., Tian S. (2020). Ca^2+^-Dependent NOX5 (NADPH Oxidase 5) Exaggerates Cardiac Hypertrophy Through Reactive Oxygen Species Production. Hypertension.

[32] Rajaram R.D., Dissard R., Jaquet V., de Seigneux S. (2019). Potential benefits and harms of NADPH oxidase type 4 in the kidneys and cardiovascular system. Nephrol. Dial. Transplant..

[33] Varga Z.V., Pipicz M., Baán J.A., Baranyai T., Koncsos G., Leszek P., Kuśmierczyk M., Sánchez-Cabo F., García-Pavía P., Brenner G.J. (2017). Alternative Splicing of NOX4 in the Failing Human Heart. Front. Physiol..

[34] Kuroda J., Ago T., Matsushima S., Zhai P., Schneider M.D., Sadoshima J. (2010). NADPH oxidase 4 (Nox4) is a major source of oxidative stress in the failing heart. Proc. Natl. Acad. Sci. USA.

[35] Rana M., Setia M., Suvas P.K., Chakraborty A., Suvas S. (2022). Diphenyleneiodonium Treatment Inhibits the Development of Severe Herpes Stromal Keratitis Lesions. J. Virol..

[36] Almeida C.R., Lima J.F., Machado M.R., Alves J.V., Couto A.E.S., Campos L.C.B., Avila-Mesquita C.D., Auxiliadora-Martins M., Becari C., Louzada-Júnior P. (2023). Inhibition of IL-6 signaling prevents serum-induced umbilical cord artery dysfunction from patients with severe COVID-19. Am. J. Physiol. Integr. Comp. Physiol..

[37] Kato Y., Nishiyama K., Man Lee J., Ibuki Y., Imai Y., Noda T., Kamiya N., Kusakabe T., Kanda Y., Nishida M. (2022). TRPC3-Nox2 Protein Complex Formation Increases the Risk of SARS-CoV-2 Spike Protein-Induced Cardiomyocyte Dysfunction through ACE2 Upregulation. Int. J. Mol. Sci..

[38] Damiano S., Sozio C., La Rosa G., Santillo M. (2020). NOX-Dependent Signaling Dysregulation in Severe COVID-19: Clues to Effective Treatments. Front. Cell. Infect. Microbiol..

[39] Zhu L., Fu X., Yuan C., Jiang X., Zhang G. (2018). Induction of Oxidative DNA Damage in Bovine Herpesvirus 1 Infected Bovine Kidney Cells (MDBK Cells) and Human Tumor Cells (A549 Cells and U2OS Cells). Viruses.

[40] Choi D.H., Lee K.H., Kim J.H., Seo J.H., Kim H.Y., Shin C.Y., Han J.S., Han S.H., Kim Y.S., Lee J. (2014). NADPH oxidase 1, a novel molecular source of ROS in hippocampal neuronal death in vascular dementia. Antioxid. Redox Signal..

[41] Sun J., Hu C., Zhu Y., Sun R., Fang Y., Fan Y., Xu F. (2015). LMP1 Increases Expression of NADPH Oxidase (NOX) and Its Regulatory Subunit p22 in NP69 Nasopharyngeal Cells and Makes Them Sensitive to a Treatment by a NOX Inhibitor. PLoS ONE.

[42] Hebchen D.M., Schader T., Spaeth M., Müller N., Graumann J., Schröder K. (2024). NoxO1 regulates EGFR signaling by its interaction with Erbin. Redox Biol..

[43] Hebchen D.M., Spaeth M., Müller N., Schröder K. (2024). NoxO1 Determines the Level of ROS Formation by the Nox1-Centered NADPH Oxidase. Antioxidants.

[44] Benssouina F.Z., Parat F., Villard C., Leloup L., Garrouste F., Sabatier J.M., Ferhat L., Kovacic H. (2023). Overexpression of a Novel Noxo1 Mutant Increases Ros Production and Noxo1 Relocalisation. Int. J. Mol. Sci..

[45] Stevenson M.D., Vendrov A.E., Yang X., Chen Y., Navarro H.A., Moss N., Runge M.S., Arendshorst W.J., Madamanchi N.R. (2023). Reactivity of renal and mesenteric resistance vessels to angiotensin II is mediated by NOXA1/NOX1 and superoxide signaling. Am. J. Physiol. Physiol..

[46] Vendrov A.E., Stevenson M.D., Lozhkin A., Hayami T., Holland N.A., Yang X., Moss N., Pan H., Wickline S.A., Stockand J.D. (2022). Renal NOXA1/NOX1 Signaling Regulates Epithelial Sodium Channel and Sodium Retention in Angiotensin II-induced Hypertension. Antioxid. Redox Signal..

[47] Eid S.A., Savelieff M.G., Eid A.A., Feldman E.L. (2022). Nox, Nox, Are You There? The Role of NADPH Oxidases in the Peripheral Nervous System. Antioxid. Redox Signal..

[48] Rousset F., Nacher-Soler G., Kokje V.B.C., Sgroi S., Coelho M., Krause K.H., Senn P. (2022). NADPH Oxidase 3 Deficiency Protects From Noise-Induced Sensorineural Hearing Loss. Front. Cell Dev. Biol..

[49] Nocella C., D’Amico A., Cammisotto V., Bartimoccia S., Castellani V., Loffredo L., Marini L., Ferrara G., Testa M., Motta G. (2023). Structure, Activation, and Regulation of NOX2: At the Crossroad between the Innate Immunity and Oxidative Stress-Mediated Pathologies. Antioxidants.

[50] Kračun D., Lopes L.R., Cifuentes-Pagano E., Pagano P.J. (2025). NADPH oxidases: Redox regulation of cell homeostasis and disease. Physiol. Rev..

[51] Herb M. (2024). NADPH Oxidase 3: Beyond the Inner Ear. Antioxidants.

[52] Buvelot H., Jaquet V., Krause K.H. (2019). Mammalian NADPH Oxidases. Methods Mol. Biol..

[53] Then A.A., Goenawan H., Lesmana R., Christoper A., Sylviana N., Gunadi J.W. (2024). Exploring the potential regulation of DUOX in thyroid hormone-autophagy signaling via IGF-1 in the skeletal muscle (Review). Biomed. Rep..

[54] Taylor J.P., Tse H.M. (2021). The role of NADPH oxidases in infectious and inflammatory diseases. Redox Biol..

[55] Zainuddin A., Hidayat I.W., Kurnia D., Ramadhanty Z.F., Padilah R. (2022). Prediction of the mechanism of action of catechin as superoxide anion antioxidants and natural antivirals for COVID-19 infection with in silico study. J. Adv. Pharm. Technol. Res..

[56] Boudreau H.E., Emerson S.U., Korzeniowska A., Jendrysik M.A., Leto T.L. (2009). Hepatitis C virus (HCV) proteins induce NADPH oxidase 4 expression in a transforming growth factor beta-dependent manner: A new contributor to HCV-induced oxidative stress. J. Virol..

[57] Chida T., Watanabe S., Ohta K., Noritake H., Ito M., Suzuki T., Suda T., Kawata K. (2024). Impact of amino acid substitutions in hepatitis C virus core region on the severe oxidative stress. Free Radic. Biol. Med..

[58] de Mochel N.S., Seronello S., Wang S.H., Ito C., Zheng J.X., Liang T.J., Lambeth J.D., Choi J. (2010). Hepatocyte NAD(P)H oxidases as an endogenous source of reactive oxygen species during hepatitis C virus infection. Hepatology.

[59] Liao T.L., Liu H.J., Chen D.Y., Tang K.T., Chen Y.M., Liu P.Y. (2023). SARS-CoV-2 primed platelets-derived microRNAs enhance NETs formation by extracellular vesicle transmission and TLR7/8 activation. Cell Commun. Signal..

[60] Naidoo P., Naidoo R.N., Ramkaran P., Muttoo S., Asharam K., Chuturgoon A.A. (2019). Maternal miRNA-146a G/C rs2910164 variation, HIV/AIDS and nitrogen oxide pollution exposure collectively affects foetal growth. Hum. Exp. Toxicol..

[61] Cho S.Y., Kim S., Son M.J., Kim G., Singh P., Kim H.N., Choi H.G., Yoo H.J., Ko Y.B., Lee B.S. (2019). Dual oxidase 1 and NADPH oxidase 2 exert favorable effects in cervical cancer patients by activating immune response. BMC Cancer.

[62] Chen G., Hao H., Ai J.W. (2018). Regulatory role of CDX2 and NOX4 expression associated with recurrent nasopharyngeal carcinoma. Eur. Rev. Med. Pharmacol. Sci..

[63] Singh G., Singh K., Sinha R.A., Singh A., Khushi Kumar A. (2024). Japanese encephalitis virus infection causes reactive oxygen species-mediated skeletal muscle damage. Eur. J. Neurosci..

[64] Jia R., Dai X., Li Y., Yang X., Min X., Quan D., Liu P., Huang X., Ge J., Ren Q. (2023). Duox mediated ROS production inhibited WSSV replication in Eriocheir sinensis under short-term nitrite stress. Aquat. Toxicol..

[65] Gingerich A., Pang L., Hanson J., Dlugolenski D., Streich R., Lafontaine E.R., Nagy T., Tripp R.A., Rada B. (2016). Hypothiocyanite produced by human and rat respiratory epithelial cells inactivates extracellular H1N2 influenza A virus. Inflamm. Res..

[66] Grandvaux N., Mariani M., Fink K. (2015). Lung epithelial NOX/DUOX and respiratory virus infections. Clin. Sci..

[67] Zhang B., Hou J., Liu J., He J., Gao Y., Li G., Ma T., Lv X., Dong L., Yang W. (2025). Hydrogen decreases susceptibility to AngII-induced atrial fibrillation and atrial fibrosis via the NOX4/ROS/NLRP3 and TGF-β1/Smad2/3 signaling pathways. PLoS ONE.

[68] Schiffer T.A., Carvalho L.R.R.A., Guimaraes D., Boeder A., Wikström P., Carlström M. (2024). Specific NOX4 Inhibition Preserves Mitochondrial Function and Dampens Kidney Dysfunction Following Ischemia-Reperfusion-Induced Kidney Injury. Antioxidants.

[69] Lv J., Shi S., Fu Z., Wang Y., Duan C., Hu S., Wu H., Zhang B., Li Y., Song Q. (2024). Exploring the inflammation-related mechanisms of Lingguizhugan decoction on right ventricular remodeling secondary to pulmonary arterial hypertension based on integrated strategy using UPLC-HRMS, systems biology approach, and experimental validation. Phytomedicine.

[70] Sofiullah S.S.M., Murugan D.D., Muid S.A., Seng W.Y., Kadir S.Z.S.A., Abas R., Ridzuan N.R.A., Zamakshshari N.H., Woon C.K. (2023). Natural Bioactive Compounds Targeting NADPH Oxidase Pathway in Cardiovascular Diseases. Molecules.

[71] Kura B., Pavelkova P., Kalocayova B., Pobijakova M., Slezak J. (2024). MicroRNAs as Regulators of Radiation-Induced Oxidative Stress. Curr. Issues Mol. Biol..

[72] Mao L., Zuo M.L., Wang A.P., Tian Y., Dong L.C., Li T.M., Kuang D.B., Song G.L., Yang Z.B. (2020). Low expression of miR5323p contributes to cerebral ischemia/reperfusion oxidative stress injury by directly targeting NOX2. Mol. Med. Rep..

[73] Lew W.Y., Bayna E., Dalle Molle E., Contu R., Condorelli G., Tang T. (2014). Myocardial fibrosis induced by exposure to subclinical lipopolysaccharide is associated with decreased miR-29c and enhanced NOX2 expression in mice. PLoS ONE.

[74] Hu Z.Y., Yang Z.B., Zhang R., Luo X.J., Peng J. (2023). The Protective Effect of Vitexin Compound B-1 on Rat Cerebral I/R Injury through a Mechanism Involving Modulation of miR-92b/NOX4 Pathway. CNS Neurol. Disord. Drug Targets.

[75] Liu Z., Tuo Y.H., Chen J.W., Wang Q.Y., Li S., Li M.C., Dai G., Wang J.S., Zhang Y.L., Feng L. (2017). NADPH oxidase inhibitor regulates microRNAs with improved outcome after mechanical reperfusion. J. NeuroInterv.l Surg..

[76] Noei Razliqi R., Ahangarpour A., Mard S.A., Khorsandi L. (2023). Gentisic acid protects against diabetic nephropathy in Nicotinamide-Streptozotocin administered male mice by attenuating oxidative stress and inflammation: The role of miR-200a/Keap1/Nrf2 pathway, renin-angiotensin system (RAS) and NF-кB. Chem. Biol. Interact..

[77] Lee M.S., Yip H.K., Yang C.C., Chiang J.Y., Huang T.H., Li Y.C., Chen K.H., Sung P.H. (2021). Overexpression of miR-19a and miR-20a in iPS-MSCs preserves renal function of chronic kidney disease with acute ischaemia-reperfusion injury in rat. J. Cell. Mol. Med..

[78] Sarkar S., Alhasson F., Kimono D., Albadrani M., Seth R.K., Xiao S., Porter D.E., Scott G.I., Brooks B., Nagarkatti M. (2020). Microcystin exposure worsens nonalcoholic fatty liver disease associated ectopic glomerular toxicity via NOX-2-MIR21 axis. Environ. Toxicol. Pharmacol..

[79] Alhasson F., Seth R.K., Sarkar S., Kimono D.A., Albadrani M.S., Dattaroy D., Chandrashekaran V., Scott G.I., Raychoudhury S., Nagarkatti M. (2018). High circulatory leptin mediated NOX-2-peroxynitrite-miR21 axis activate mesangial cells and promotes renal inflammatory pathology in nonalcoholic fatty liver disease. Redox Biol..

[80] Nazari B., Jaquet V., Krause K.H. (2023). NOX family NADPH oxidases in mammals: Evolutionary conservation and isoform-defining sequences. Redox Biol..

[81] D’Aloja P., Olivetta E., Bona R., Nappi F., Pedacchia D., Pugliese K., Ferrari G., Verani P., Federico M. (1998). gag, vif, and nef genes contribute to the homologous viral interference induced by a nonproducer human immunodeficiency virus type 1 (HIV-1) variant: Identification of novel HIV-1-inhibiting viral protein mutants. J. Virol..

[82] Al-Muffti A.S., Kiarie I.W., Tőzsér J., Mahdi M. (2025). Modulation of transferrin receptor by HIV-2. Virol. J..

[83] Biryukov J., Meyers C. (2018). Superinfection Exclusion between Two High-Risk Human Papillomavirus Types during a Coinfection. J. Virol..

[84] Shi D., Zhao Y., Zhao X., Gong Z., Liu W., Li P., Zhang Y., Luo B. (2026). Epstein-Barr Virus Hijacks Redox Signaling via Glutathione Peroxidase 4 to Sustain Latency and Drive Gastric Cancer Progression. Antioxid. Redox Signal..

